# Carbon fibre cloth-supported iridium oxide/polyaniline bilayer electrode for solid-state potentiometric pH sensing

**DOI:** 10.1039/d6ra05424b

**Published:** 2026-07-23

**Authors:** Md Rasel, Mamun Jamal, N. Padmanathan, Kafil M. Razeeb

**Affiliations:** a Department of Chemistry, Khulna University of Engineering & Technology Khulna 9203 Bangladesh mamun.jamal@chem.kuet.ac.bd; b Micro-NanoSystems Centre, Tyndall National Institute, University College Cork Lee Maltings Complex, Dyke Parade Cork T12 R5CP Ireland kafil.mahmood@tyndall.ie

## Abstract

In this work, a stable solid-state potentiometric pH sensor was developed by integrating an iridium oxide/polyaniline (IrO_2_/PANI) bilayer onto conductive carbon fibre cloth (CFC). The resulting IrO_2_/PANI/CFC electrode was fabricated through sequential PANI electropolymerization followed by IrO_2_ electrodeposition, forming a binder free hybrid interface that combines the proton responsive conductivity of PANI with hydrated/hydroxylated proton active iridium oxide sites. Morphological, elemental, vibrational, and surface chemical analyses confirmed the successful formation of the IrO_2_/PANI sensing layer on the CFC substrate. The electrode exhibited a near-Nernstian response over pH 3–11, with a sensitivity of 58.4 ± 0.9 mV pH^−1^, good linearity and an estimated pH resolution of approximately 0.01 pH unit. During continuous pH adjustment in Britton–Robinson buffer from pH 4.5 to 11.1, the electrode maintained a comparable sensitivity of 56.8 ± 1.1 mV pH^−1^ without removing or washing the sensor. The electrode also showed limited interference from common cations, reversible pH tracking behaviour, acceptable short-term drift and 96.3% sensitivity retention after 45 days of repeated measurement and storage. Real-sample analysis of vinegar, orange juice, and baking powder solution showed good agreement with a commercial glass membrane pH meter. These results demonstrate the potential of IrO_2_/PANI/CFC as a low-cost platform for stable potentiometric pH sensing in practical sample environments.

## Abstract 2

In this work, a stable solid-state potentiometric pH sensor was developed by integrating a bilayer of iridium oxide (IrO_2_) and polyaniline (PANI) onto conductive carbon fiber cloth (CFC).

## Graphical abstract

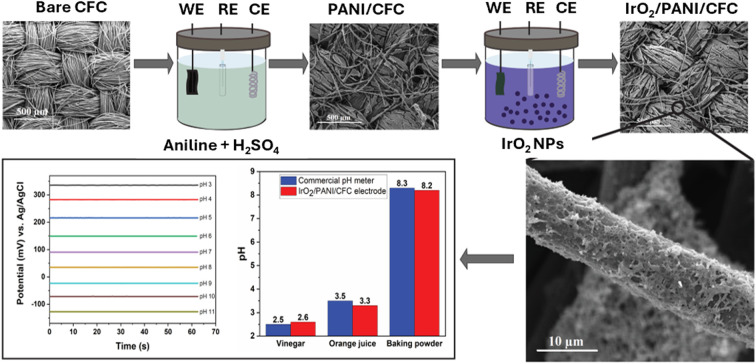

## 1 Introduction

pH is one of the most important chemical parameters for monitoring aqueous systems in environmental analysis, food quality control, biomedical diagnostics, agriculture and industrial processing.^1–3^ Accurate pH determination is essential because small changes in proton concentration can significantly affect chemical reactions, biological activity, corrosion behaviour, and product quality.^1,4^ Conventional glass electrodes remain the most widely used pH sensors due to their high proton selectivity and reliable potentiometric response. However, their intrinsic fragility, relatively high internal resistance, susceptibility to membrane fouling, and limited compatibility with miniaturized or deformable platforms restrict their use in miniaturized, field-deployable, and non-rigid sensing systems.^5,6^ Recent pH-monitoring strategies have expanded beyond conventional glass electrodes to include optical, spectroscopic and solid-state electrochemical platforms. In particular, surface enhanced Raman scattering (SERS) based probes, including hydrogel-isolated dual-carboxyl SERS probes, SERS microtips and reproducible SERS sensors for biological pH detection, have enabled sensitive pH readout in localized or microenvironmental conditions.^7–9^ Although these optical approaches offer high sensitivity and spatially resolved detection, they usually require plasmonic substrates and optical instrumentation. These considerations highlight the need for solid-state potentiometric pH sensors that are low-cost, mechanically robust and stable, while retaining the simplicity of direct electrical readout for practical aqueous sample analysis.

Mechanically compliant conductive substrates provide an attractive route for replacing rigid glass-based pH electrodes and developing solid-state electrochemical sensors with direct electrical readout.^10^ Recent studies on binder-free electrodes, carbon-based fabrics, polymer/nanocomposite interfaces, metal oxide/carbon hybrids and microtextured sensing interfaces have shown that stable sensor performance depends not only on the active sensing material but also on efficient charge transport, interfacial contact and integration with the supporting substrate.^11–14^ Among conductive substrates, carbon fibre cloth (CFC) is particularly promising because of its high electrical conductivity, chemical stability, mechanical flexibility, porous structure, and low cost.^15,16^ In addition, CFC can serve directly as a current collector without requiring additional binders or conductive additives.^17^ However, pristine CFC is not intrinsically proton sensitive and therefore its surface must be functionalized with an active pH responsive material to produce a reliable potentiometric response.

Conducting polymers have been widely explored as proton-sensitive layers for solid-state pH sensors. Polyaniline (PANI) is especially attractive because of its facile synthesis, low cost, environmental stability, reversible protonation/deprotonation behaviour, and good electrical conductivity.^18,19^ Recently, Hossain *et al.* demonstrated PANI modified CFC as an effective flexible pH sensing platform, highlighting the potential of CFC supported conducting polymers for solid state potentiometric sensing.^20^ However, PANI-based electrodes may still suffer from structural instability, swelling/shrinkage during repeated protonation–deprotonation cycles, and potential drift during long-term operation.^21,22^ These limitations can reduce reproducibility and operational stability, particularly when the sensor is used continuously or in complex real samples.

Metal oxides offer an alternative class of robust pH sensitive materials due to their proton coupled redox activity, chemical stability, and wide pH operating range.^2,23^ Several metal oxides, including TiO_2_, RuO_2_, Ta_2_O_5_, SnO_2_, and IrO_2_, have been investigated for solid state pH sensing.^2,24,25^ Among these, iridium oxide is considered one of the most reliable pH sensitive oxides because of its fast and reversible proton exchange, near-Nernstian potentiometric response, and high chemical stability in acidic and alkaline environments.^26,27^ However, only metal oxide sensing layers may suffer from limited mechanical compliance and reduced interfacial contact when integrated with flexible or porous substrates.^28^

Combining conducting polymers with metal oxides is a promising strategy to overcome the limitations of each individual component in solid-state pH sensing. In such hybrid systems, the conducting polymer provides a conductive and mechanically accommodating pathway for charge transport, while the metal oxide contributes chemically stable proton active sites for potentiometric transduction.^29,30^ This strategy has also been explored for pH sensing; for example, a PANI-Ni(OH)_2_ modified nickel foam electrode recently showed a linear response over pH 3–11 with a sensitivity of 46.00 mV pH^−1^, although the response remained below the near-Nernstian value and multi-day long-term sensitivity retention was not demonstrated.^31^ This comparison indicates that further development of stable polymer/oxide pH-sensing interfaces remains important. On this basis, integrating IrO_2_/IrO_*x*_ with PANI on CFC is expected to create a complementary sensing interface: PANI improves electrical connectivity and surface coverage on the porous carbon fibre network, whereas IrO_2_/IrO_*x*_ contributes hydrated/hydroxylated proton-active sites for pH-dependent potential generation. Recently, Lee *et al.* reported a long-term stable PANi-IrO_*x*_ bilayer pH sensor array for real time two-dimensional pH mapping in cell culture monitoring, demonstrating the benefit of polymer/oxide bilayer architectures.^32^ However, that device was based on a microfabricated polyimide/Cu/Au platform, whereas a low-cost, binder free IrO_2_/PANI bilayer directly on CFC for practical food and environmental pH monitoring remains less explored.

Herein, we report a binder free IrO_2_/PANI bilayer electrode fabricated directly on carbon fibre cloth for solid state potentiometric pH sensing. The architecture integrates the conductive and proton responsive nature of PANI with the chemically stable proton active redox sites of IrO_2_, while CFC serves as a flexible, porous, and conductive platform. The proposed design provides a simple and practical platform for low-cost flexible pH sensors intended for environmental and food sample monitoring.

## 2 Experimental

### 2.1 Materials

Potassium hexachloroiridate(iv), hydrochloric acid, sulfuric acid, aniline and ethanol were purchased from E. Merck, Germany. Sodium carbonate, sodium bicarbonate, disodium hydrogen phosphate, sodium dihydrogen phosphate and sodium hydroxide pellets were obtained from Sigma-Aldrich. Carbon fibre cloth was purchased from radionics Ireland. Copper conducting wire and insulating polymer were used for electrical connection and electrode encapsulation respectively. Malt vinegar, orange juice, and baking powder were purchased from local markets in Bangladesh and used as real samples. All chemicals were of analytical grade and used without further purification. All aqueous solutions were prepared using ultrapure Millipore water with a resistivity of approximately 18.2 MΩ cm.

### 2.2 Synthesis of IrO_2_ nanoparticles

IrO_2_ nanoparticles were synthesized using a previously reported chemical method with slight modification.^33^ Briefly, 10 wt% NaOH was added dropwise to a 2 mM aqueous K_2_IrCl_6_ solution under continuous stirring until the solution became strongly alkaline. The solution changed to yellow, indicating hydrolysis of the iridium precursor. The mixture was then heated at 90 °C for 1 h under continuous stirring, during which the colour gradually changed to light blue. The solution was immediately cooled in an ice bath, and 3 M HNO_3_ was added dropwise until the solution became acidic. The resulting dark blue IrO_2_ nanoparticle dispersion was stirred for 80 min at room temperature and then aged for 24 h at 4 °C in the dark. The colloidal IrO_2_ dispersion was stored at 4 °C and brought to room temperature before use.

### 2.3 Fabrication of working electrode

**Scheme 1:**
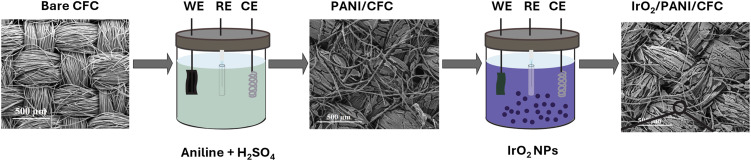
Schematic illustration of the fabrication route of the IrO_2_/PANI/CFC pH sensor.

As summarized in Scheme 1, the IrO_2_/PANI/CFC electrode was prepared by sequential modification of CFC through PANI electropolymerization followed by IrO_*x*_ nanoparticle electrodeposition. CFC pieces with an approximate size of 1 × 1 cm^2^ were ultrasonically cleaned in 3 M HCl for 30 min, followed by ethanol for 30 min, to remove surface impurities. The cleaned CFC was rinsed thoroughly with deionized water and dried at room temperature. Electrical contact was made using copper wire and the contact region was sealed with an inert insulating polymer to define the active electrode area. Polyaniline was electropolymerized on the cleaned CFC substrate in a three-electrode cell containing 0.1 M aniline and 1.0 M H_2_SO_4_. The CFC substrate was used as the working electrode, a platinum wire as the counter electrode, and Ag/AgCl (3 M KCl) as the reference electrode. Electropolymerization was performed by cyclic voltammetry for 10 cycles over a potential window of −0.2 to 0.8 V at a scan rate of 20 mV s^−1^; these conditions were selected based on our group's previously reported PANI/CFC pH sensor.^20^ The resulting PANI/CFC electrode was rinsed several times with deionized water to remove residual monomer and electrolyte and then air-dried for 6 h at room temperature. Then, the pre-synthesized IrO_*x*_ nanoparticles were deposited onto the PANI/CFC by electrochemically assisted anodic deposition. During the electrodeposition procedure, PANI/CFC was exposed to a potential of +1.4 V against Ag/AgCl for 30 minutes in a 20 mL IrO_*x*_ NPs solution.^34^ Before conducting electrochemical experiments, the sample was rinsed with water and air-dried at room temperature.

### 2.4 Material characterization and potentiometric pH measurements

Electrochemical measurements were performed using a potentiostat/galvanostat (Biologic SP-300). A conventional three-electrode configuration was used, consisting of the IrO_2_/PANI/CFC electrode as the working electrode, Ag/AgCl (3 M KCl) as the reference electrode, and a platinum wire as the counter electrode. Open-circuit potential measurements were carried out in buffer solutions of different pH values to evaluate the potentiometric pH response. The surface morphology and elemental composition of the electrodes were examined using field-emission scanning electron microscopy (FE-SEM, FEI Quanta 650) equipped with energy-dispersive X-ray spectroscopy (EDX, Oxford Instruments INCA energy system). Fourier-transform infrared spectroscopy (FTIR) spectra were recorded using an IRTracer-100 spectrometer (Shimadzu). Raman spectra were collected using a Renishaw RA100 confocal Raman microscope with a 514.5 nm excitation laser. X-ray photoelectron spectroscopy (XPS) was performed using a Kratos AXIS ULTRA spectrometer. UV-visible absorption spectra of the IrO_*x*_ dispersion were recorded using a Shimadzu UV-1800 spectrophotometer in the wavelength range of 200–800 nm using a quartz cuvette.

## 3 Results and discussion

### 3.1 Fabrication of pH sensor

**Fig. 1:**
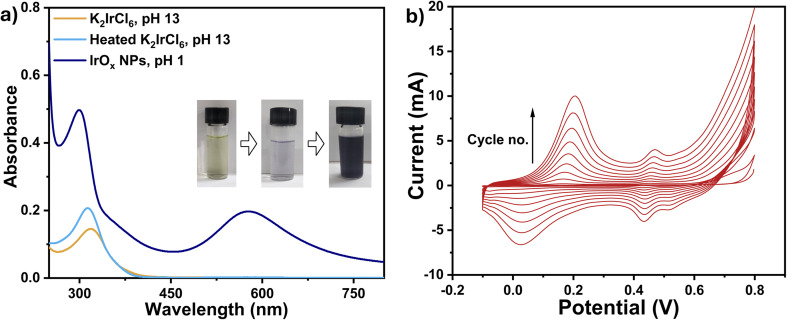
(a) UV-vis spectra showing the formation of colloidal IrO_*x*_ nanoparticles from K_2_IrCl_6_ precursor solution. The inset shows the corresponding colour transition during IrO_*x*_ formation; (b) cyclic voltammograms recorded during electropolymerization of PANI on CFC in 0.1 M aniline and 1.0 M H_2_SO_4_; the arrow indicates increasing cycle number.

The formation of IrO_2_/PANI/CFC electrode was systematically examined at each fabrication stage, beginning with the synthesis of colloidal IrO_*x*_ nanoparticles and the electropolymerization of PANI on CFC. The formation of colloidal IrO_*x*_ nanoparticles was monitored by UV-vis spectroscopy. As shown in Fig. 1a, the alkaline K_2_IrCl_6_ precursor solution exhibited an absorption feature around 313 nm, which can be attributed to hydrolysis of the Ir–Cl coordination environment and formation of hydrolysed iridium species.^33,35^ Upon heating at 90 °C, hydrolysis of [IrCl_6_]^2−^ is promoted, leading to the formation of [Ir(OH)_6_]^2−^ type species. Subsequent acidification induces protonation and condensation of the hydrolysed iridium species, resulting in the formation of Ir–O–Ir networks in colloidal hydrated iridium oxide. This transformation was confirmed by the appearance of a broad absorption band around 580 nm, which is characteristic of IrO_*x*_·*n*H_2_O nanoparticles.^33^ The corresponding colour transition from pale yellow to light blue and finally to dark blue, shown in the inset of Fig. 1a, further supports the formation of colloidal IrO_*x*_ nanoparticles.

PANI was then electropolymerized directly on the CFC substrate by cyclic voltammetry in acidic aniline solution. As shown in Fig. 1b, the cyclic voltammograms show the characteristic redox behaviour of PANI, associated with reversible transitions among leucoemeraldine, emeraldine, and pernigraniline oxidation states.^36,37^ The gradual increase in anodic and cathodic current with increasing cycle number indicates the progressive formation of an electroactive PANI layer on the CFC surface. Since the electropolymerization was performed in strongly acidic medium, the deposited PANI is expected to be in its protonated conductive form, which is favourable for charge transport and proton-responsive sensing.^36,38^ Finally, the pre-formed IrO_*x*_ colloidal nanoparticles were electrodeposited onto the PANI/CFC electrode at +1.40 V *versus* Ag/AgCl for 1800 s to obtain the IrO_2_/PANI/CFC sensing electrode.^34^ This step is more appropriately described as electrochemically assisted anodic colloidal deposition rather than conventional electrodeposition from dissolved iridium ions because pre-formed IrO_*x*_ colloidal species were used. The applied anodic potential is expected to promote transport and accumulation of the colloidal species at the PANI/CFC surface, followed by their aggregation and attachment to the porous polymer layer.^35^ The successful formation of the hybrid sensing layer was further confirmed by morphological, elemental, and surface chemical analyses, as discussed in the following sections.

### 3.2 Morphology characterization of electrodes

**Fig. 2:**
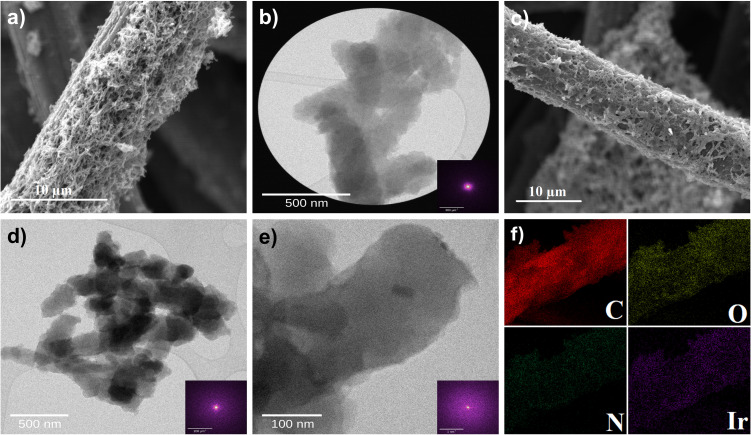
(a) SEM and (b) TEM images of PANI/CFC; (c) SEM, (d) TEM and (e) high-magnification TEM image of IrO_2_/PANI/CFC with FFT inset of IrO_2_/PANI/CFC; (f) EDX elemental maps of IrO_2_/PANI/CFC showing C, N, O, and Ir distributions, respectively.

The morphology and elemental distribution of the modified electrodes were investigated by SEM, TEM and EDX elemental mapping to verify the formation of the IrO_2_/PANI hybrid sensing layer on CFC. The bare CFC showed a woven carbon fibre network with relatively smooth fibre surfaces, as presented in Fig. S1. After electropolymerization, a clear morphological transformation was observed. As shown in Fig. 2a, the PANI/CFC electrode exhibited a rough and porous coating along the carbon fibre surface, confirming the successful growth of PANI on the flexible CFC substrate. The interconnected PANI network provides an enlarged accessible interface and continuous conductive pathways, which are beneficial for proton exchange and charge transport during potentiometric pH sensing.^20,39^

The corresponding TEM image in Fig. 2b shows a semi-transparent polymeric morphology, consistent with the amorphous and low electron density nature of electropolymerized PANI. After IrO_*x*_ electrodeposition, the surface became more heterogeneous and densely textured. Fig. 2c shows that the IrO_2_/PANI/CFC electrode retained the porous fibre supported architecture, while additional nanoscale features appeared on the PANI coated surface. This morphology indicates that the iridium oxide phase was incorporated into or deposited onto the PANI network without disrupting the flexible CFC framework. Further nanoscale evidence is provided by the TEM images of IrO_2_/PANI/CFC. As shown in Fig. 2d, darker contrast domains are distributed within the lighter PANI based matrix. These darker regions are attributed to Ir containing oxide species because iridium oxide has higher electron density than the organic PANI phase. The high-magnification TEM image in Fig. 2e further reveals nanoscale IrO_*x*_ rich regions within the hybrid structure, supporting the formation of an intimately integrated IrO_*x*_/PANI interface.^32^

The chemical composition and spatial distribution of these IrO_*x*_ rich regions were further evaluated by EDX elemental mapping. For PANI/CFC, the EDX elemental maps and spectrum in Fig. S2 show C and N distributed over the modified carbon fibre surface, supporting the formation of the PANI coating. An O signal is also observed, which can be attributed to native oxygen containing groups on the carbon fibre surface and/or adsorbed oxygen/water.^40^ After IrO_*x*_ electrodeposition, the IrO_2_/PANI/CFC electrode exhibits a clear Ir distribution in addition to C, N, and O as shown in Fig. 2f and S3. The emergence and spatial distribution of Ir over the modified surface, together with its association with O in the elemental maps, supports the successful incorporation of the iridium oxide phase into the PANI coated CFC electrode. Overall, the SEM, TEM and EDX results demonstrate that the sequential electropolymerization/electrodeposition strategy produced a porous IrO_2_/PANI hybrid interface on the flexible CFC substrate.

### 3.3 Structural and surface chemical characterization

**Fig. 3:**
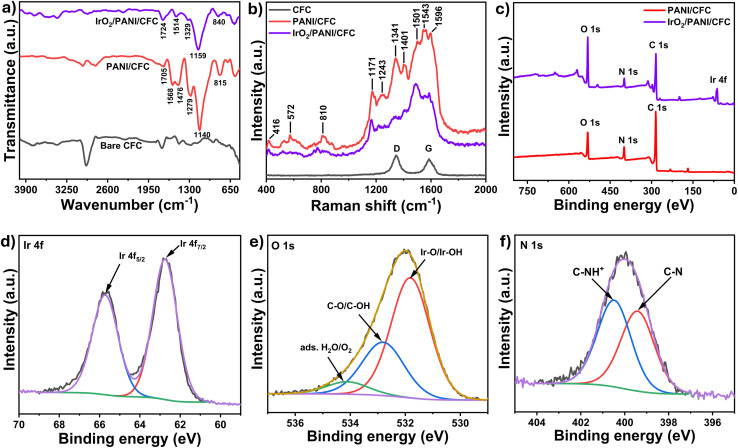
(a) FTIR spectra of CFC, PANI/CFC, and IrO_2_/PANI/CFC; (b) Raman spectra of CFC, PANI/CFC, and IrO_2_/PANI/CFC; (c) XPS survey spectra of PANI/CFC and IrO_2_/PANI/CFC; high-resolution XPS spectrum of IrO_2_/PANI/CFC: (d) Ir 4f, (e) O 1s, (f) N 1s.

FTIR, Raman spectroscopy, and XPS were used to further examine the structural and surface chemical features of the modified electrodes. The FTIR spectra in Fig. 3a show the characteristic vibrational signatures of CFC, PANI/CFC, and IrO_2_/PANI/CFC. Bare CFC exhibits weak but discernible absorption features, as expected for a graphitic carbon-based substrate. A relatively sharp band cantered at approximately 2925 cm^−1^ is attributed to aliphatic C–H stretching of methylene groups, which may originate from residual surface organic species or sizing agents on the commercial carbon fibre surface. Weak bands around 1500–1600 cm^−1^ and 1750 cm^−1^ can be assigned to C=C vibrations of graphitic carbon domains and C=O stretching of oxygen containing surface groups, respectively.^41^ After electrodeposition of PANI, several characteristic peaks appeared in the FTIR of PANI/CFC, including the C=C stretching vibrations of benzenoid and quinoid ring at 1476 cm^−1^ and 1568 cm^−1^ respectively.^42^ The broad N–H stretching vibration around 3000–3200 cm^−1^ attributed to the amine group of PANI backbone. The peak at 1140 cm^−1^ corresponds to the protonated quinoid structure of conductive PANI, which is essential for enhanced charge transfer and electrochemical activity.^43^ Additionally, the peak around 1300 cm^−1^ is assigned to C–N stretching vibrations, while peak near 815 cm^−1^ correspond to out of plane C–H bending in the PANI backbone.^44^ These features confirm the successful formation of PANI on the CFC substrate. In the IrO_2_/PANI/CFC spectrum, the characteristic PANI bands are retained, although their relative intensity changes after IrO_*x*_ deposition. This variation can be attributed to partial surface coverage by the deposited iridium oxide phase and possible interfacial interaction between IrO_*x*_ and the PANI matrix.

Raman spectroscopy was further employed to examine the vibrational features of the modified electrodes. As shown in Fig. 3b, bare CFC exhibits two characteristic carbon bands at approximately 1345 and 1586 cm^−1^, corresponding to the D and G bands, respectively.^45^ The D band is associated with structural disorder or defects in carbon, whereas the G band arises from the in-plane vibration of sp^2^ hybridized graphitic carbon domains. After PANI electropolymerization, the PANI/CFC spectrum shows several additional characteristic PANI related bands. In the low-wavenumber region, the bands at approximately 416, 530, 572, and 810 cm^−1^ can be attributed to skeletal/ring deformation modes and aromatic C–H out-of-plane bending vibrations of the PANI backbone.^18^ In the higher-wavenumber region, the bands at 1171 and 1243 cm^−1^ are assigned to aromatic C–H bending and C–N stretching vibrations of PANI, respectively. The feature near 1341 cm^−1^ is associated with C–N^+^/polaronic structures of conductive PANI, although this band overlaps with the D band of the carbon fibre substrate.^46^ The bands at 1401, 1501, 1543, and 1596 cm^−1^ are attributed to benzenoid/quinoid ring vibrations and C=N/C=C stretching modes in the PANI backbone, with the 1596 cm^−1^ band partially overlapping with the carbon G band.^47^ These Raman features support the successful formation of the PANI layer on CFC and are consistent with the FTIR results. For the IrO_2_/PANI/CFC electrode, the main PANI related Raman bands remain detectable after IrO_*x*_ electrodeposition, although their intensities are reduced because of partial surface coverage and attenuation by the deposited iridium oxide phase. No distinct new IrO_2_ Raman band is resolved, likely because of the thin, hydrated/nanostructured nature of the electrodeposited IrO_*x*_ layer and overlap with the stronger PANI/CFC background. Therefore, Raman analysis mainly supports retention of the PANI structure, while the iridium oxide phase is confirmed by XPS.

XPS analysis was performed to examine the surface elemental composition and chemical states of the modified electrodes. As shown in Fig. 3c, the survey spectrum of PANI/CFC exhibits C 1s, N 1s, and O 1s signals, confirming the presence of the PANI coated carbon framework. After IrO_*x*_ electrodeposition, the IrO_2_/PANI/CFC electrode exhibits additional Ir 4f signals, confirming the successful incorporation of an iridium-containing oxide phase onto the PANI/CFC surface. The high-resolution Ir 4f spectrum of IrO_2_/PANI/CFC in Fig. 3d shows two main peaks at approximately 62.8 and 65.7 eV, corresponding to Ir 4f_7/2_ and Ir 4f_5/2_, respectively.^48^ These binding energies are consistent with IrO_2_ like iridium oxide species, supporting the formation of the iridium oxide phase on the PANI modified CFC electrode. The assignment is further supported by the O 1s spectrum and the Ir/O distribution observed in the elemental maps. The high-resolution O 1s spectrum of IrO_2_/PANI/CFC in Fig. 3e was deconvoluted into three components at 531.8, 532.8, and 534.1 eV. The component at 531.8 eV is assigned to Ir–O/Ir–OH species associated with hydrated iridium oxide.^49^ The peak at 532.8 eV is attributed to C–O/C–OH and surface hydroxyl groups from the hybrid PANI/CFC framework, while the higher binding energy component at 534.1 eV corresponds to adsorbed water/oxygen or weakly bound surface oxygen species.^50^ These oxygen-containing and hydroxylated surface species are beneficial for proton-coupled pH sensing. The high-resolution N 1s spectrum in Fig. 3f further confirms the nitrogen-containing PANI structure within the hybrid electrode. The fitted components at 399.4 and 400.5 eV are assigned to neutral nitrogen species in the PANI backbone, mainly C–N/–NH– groups, and protonated or oxidized nitrogen species such as C–NH^+^/N^+^, respectively.^51,52^

The continued presence of the characteristic PANI features in the FTIR and Raman spectra, together with the PANI-related N 1s components in the XPS spectrum indicates that the polymer framework was largely preserved after IrO_*x*_ deposition. Nevertheless, minor surface oxidation under these strongly oxidizing conditions cannot be completely excluded. Additional C 1s fitting profiles of PANI/CFC and IrO_2_/PANI/CFC, together with the N 1s spectrum of PANI/CFC, are provided in Fig. S4 to further support the carbon and nitrogen containing PANI framework before and after IrO_*x*_ deposition. Overall, the XPS results confirm the successful formation of a PANI supported iridium oxide interface, in which PANI provides a conductive nitrogen containing matrix and iridium oxide contributes hydroxylated proton active sites for potentiometric pH transduction.

### 3.4 pH sensor performance

**Fig. 4:**
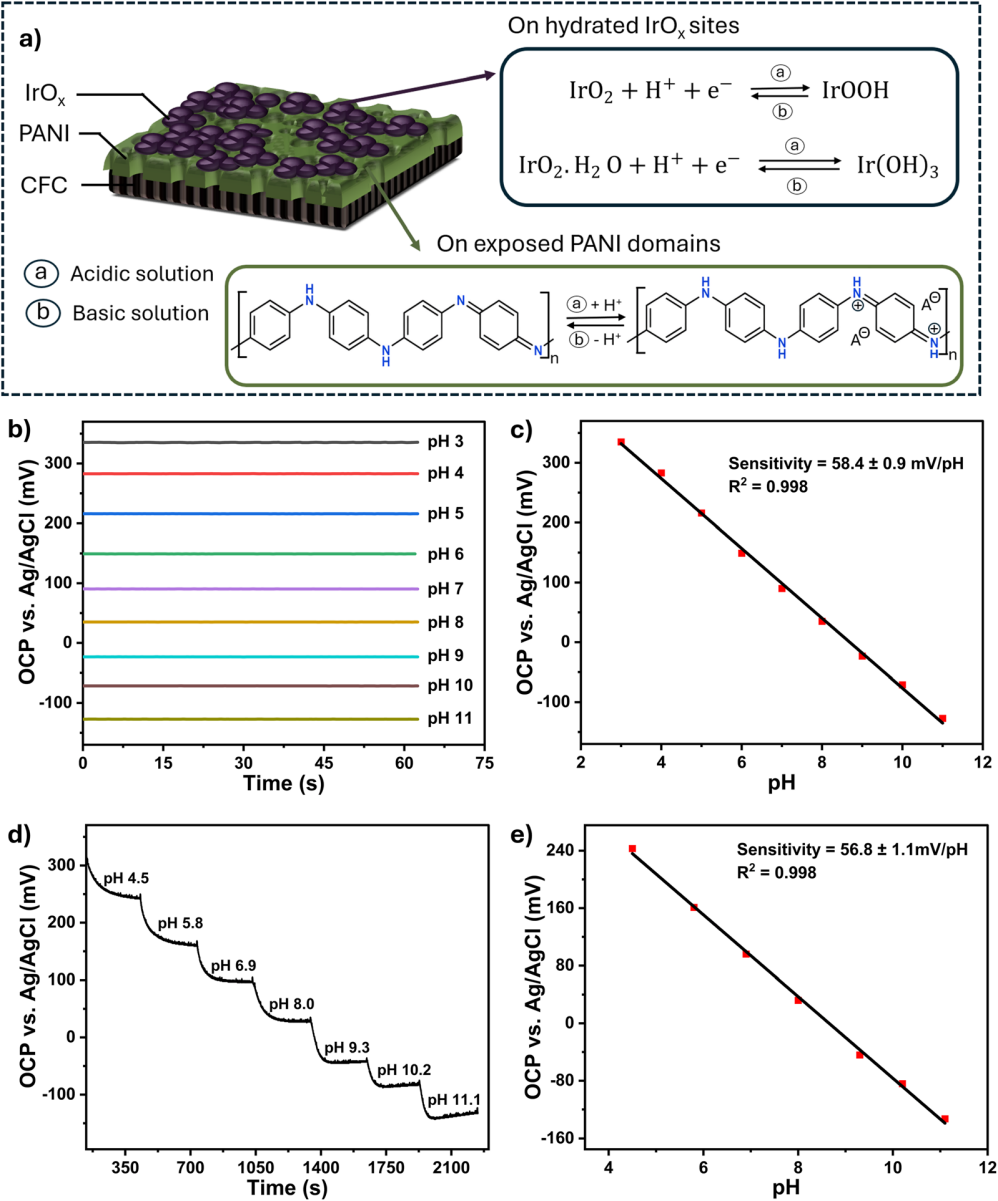
(a) Schematic illustration of the pH sensing mechanism; (b) open circuit potential response of the electrode in standard buffer solutions from pH 3 to 11 and (c) its corresponding calibration curve; (d) continuous OCP response recorded in Britton–Robinson buffer while the pH was adjusted from 4.5 to 11.1 by adding 1 M NaOH without removing or washing the sensor and (e) corresponding calibration curve extracted from the continuous pH response measurement.

The potentiometric pH sensing performance of the IrO_2_/PANI/CFC electrode was evaluated using open circuit potential measurements in standard buffer solutions. The proposed sensing mechanism is illustrated in Fig. 4a. In this hybrid architecture, the CFC substrate acts as a porous conductive support, while the PANI/IrO_2_ interface serves as the proton responsive sensing layer. The electrodeposited IrO_2_/IrO_*x*_ domains partially cover the PANI matrix, allowing both hydrated/hydroxylated iridium oxide sites and exposed PANI domains to participate in proton exchange. Changes in solution pH modulate proton coupled equilibria at hydrated IrO_2_/IrO_*x*_ sites,^53,54^ while the PANI backbone undergoes reversible protonation/deprotonation of nitrogen sites.^18^ The close contact between IrO_2_/IrO_*x*_ domains and the PANI matrix, supported by the TEM and XPS analyses, suggests that the oxide and polymer components act as a coupled proton-responsive interface rather than as independent sensing layers. These coupled interfacial processes alter the charge distribution at the electrode/electrolyte interface and support charge transfer from the IrO_2_/IrO_*x*_ sites through the conducting PANI/CFC network, resulting in an open-circuit potential that can be correlated with solution pH using an Ag/AgCl reference electrode. The near-Nernstian response can be understood from the Nernst relationship for a simplified one proton/one electron interfacial process:1
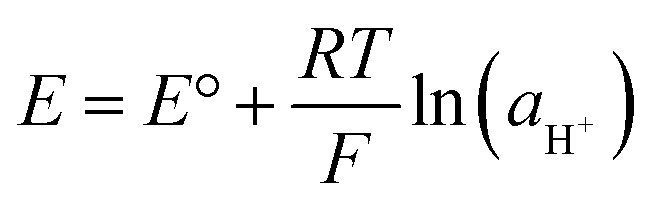
where *E*° is standard electrode potential, *R* is the molar gas constant, *T* is the absolute temperature and *F* is the Faraday constant. Since pH is related to proton activity, eqn (1) corresponds to an ideal Nernstian slope of 59.16 mV pH^−1^ at 25 °C for a one-proton/one-electron process.

As shown in Fig. 4b, the OCP was recorded for approximately 1 min in separate standard buffer solutions across the pH range of 3–11. The electrode produced stable potential profiles at each pH value, with the measured potential decreasing systematically as the pH increased. This pH dependent potential shift confirms the proton sensitive response of the IrO_2_/PANI/CFC interface. The corresponding calibration curve in Fig. 4c shows a strong linear relationship between OCP and pH, with a sensitivity of 58.4 ± 0.9 mV pH^−1^ and an *R*^2^ value of 0.998 (*n* = 3 independent electrodes). This sensitivity is close to the theoretical Nernstian response, indicating efficient proton coupled interfacial charge transfer at the hybrid electrode. The minimum detectable pH change, estimated as ΔpH = Δ*V*/*S*, was approximately 0.01 pH unit, where Δ*V* is the potential uncertainty and *S* is the calibration sensitivity. Additional cyclic voltammetric measurements were performed at pH 6, 7, and 8 to examine the electrochemical response of the IrO_2_/PANI/CFC electrode under near neutral conditions (Fig. S5a). A slight shift of the anodic feature toward lower potentials was observed with increasing pH, indicating that the electrochemical response of the hybrid interface is influenced by solution pH.

To further assess dynamic pH sensing capability, the IrO_2_/PANI/CFC electrode was tested in Britton–Robinson buffer while the solution pH was continuously adjusted from pH 4.5 to 11.1 by adding 1 M NaOH, without removing or washing the sensor between pH changes. The OCP response of the sensor and the pH value measured by a commercial pH meter were recorded simultaneously. As shown in Fig. 4d, the electrode exhibited a clear potential change following the gradual increase in pH, demonstrating its ability to monitor continuous pH variation in real confirming that the IrO_2_/PANI/CFC electrode maintains near-Nernstian behavior under continuous pH adjustment conditions.

**Fig. 5:**
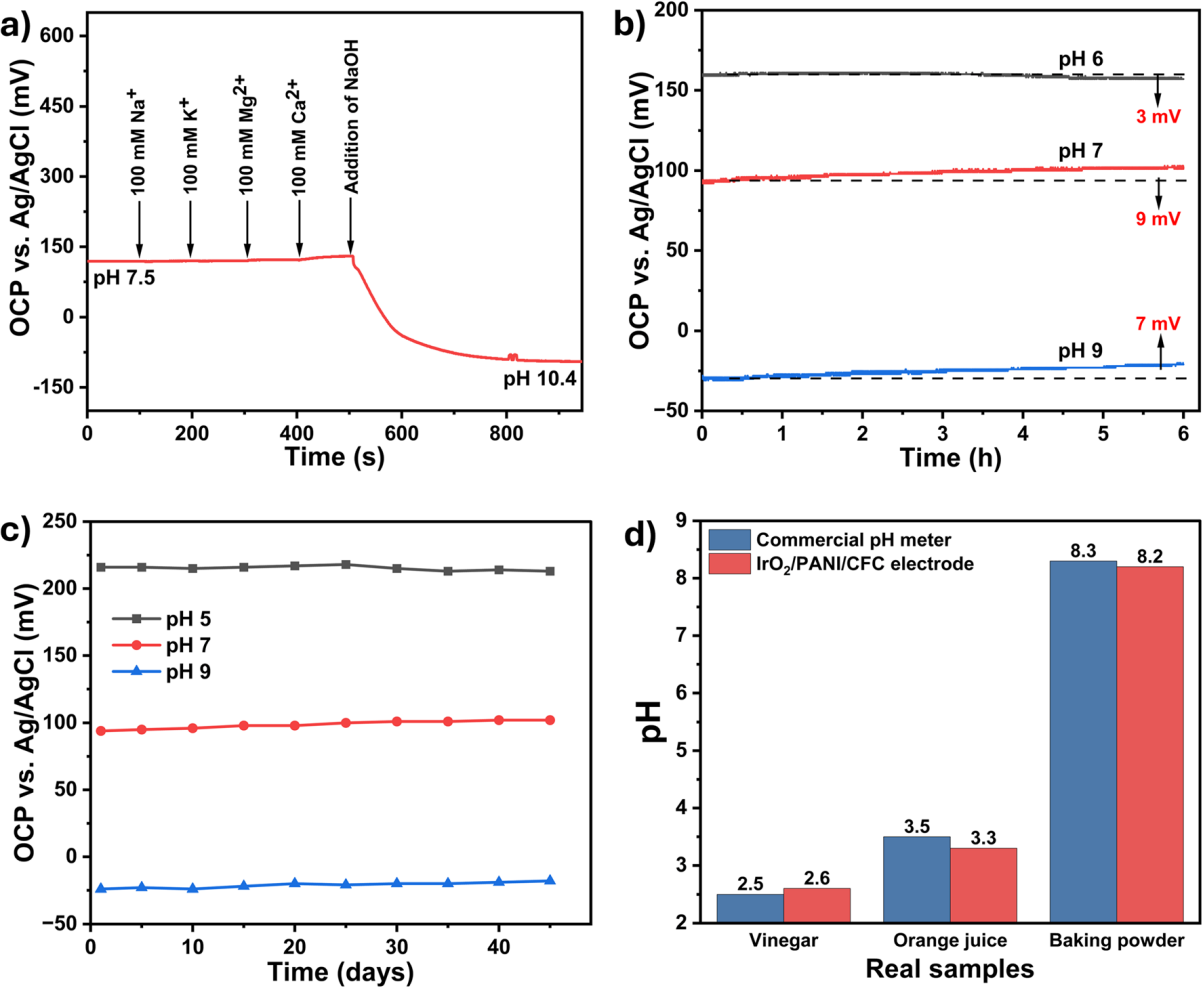
(a) Interference study in the presence of common cations, including Na^+^, K^+^, Mg^2+^ and Ca^2+^ followed by pH adjustment using NaOH; (b) short-term OCP drift response recorded at fixed pH values during 6 hours continuous measurement; (c) long-term stability of the electrode measured at pH 5, 7 and 9 over 45 days. (d) Real sample pH analysis of vinegar, orange juice and baking powder solution compared with a commercial pH meter.

The calibration curve extracted from this dynamic measurement is shown in Fig. 4e, giving a sensitivity of 56.8 ± 1.1 mV pH^−1^. This value is close to the static calibration sensitivity, the practical analytical performance of the IrO_2_/PANI/CFC electrode was further evaluated through interference, reversibility, short-term drift, long-term stability, and real sample measurements. The interference study was performed to examine whether common cations affect the pH response of the electrode. As shown in Fig. 5a, the OCP response was recorded in the presence of Na^+^, K^+^, Mg^2+^ and Ca^2+^ at 100 mM concentration. The addition of these cations caused only minor potential variations, whereas a clear potential change was observed when the solution pH was adjusted using NaOH. This result indicates that the response of the IrO_2_/PANI/CFC electrode is mainly governed by changes in proton activity rather than by the tested interfering cations. The reversibility of the electrode response was further examined through a pH titration cycle, as shown in Fig. S5b. During this test, the solution pH was changed from 6.1 to 10.4 and then to 4.1 without removing or washing the sensor between pH changes. The OCP response changed clearly with the pH variation and followed the direction of the pH change measured using a commercial pH meter. This result supports the reversible pH tracking behaviour of the IrO_2_/PANI/CFC electrode under continuous titration conditions.

The short-term operational stability of the electrode was evaluated by continuously recording the OCP at fixed pH values for 6 h. As shown in Fig. 5b, the electrode exhibited stable potential profiles during the initial stage of continuous measurement, with potential drift values of approximately 1.15, 2.0 and 2.0 mV at pH 6, 7, and 9 respectively after 1 h. After prolonged continuous exposure for 6 h, the total potential drift increased to approximately 3, 9 and 7 mV at pH 6, 7 and 9 respectively. These results indicate that the IrO_2_/PANI/CFC electrode maintains acceptable short-term stability under continuous measurement conditions. For prolonged monitoring applications, this gradual baseline shift could be managed through periodic reference correction, single-point recalibration, or software-based drift compensation. Long term stability was investigated by monitoring the OCP response of the electrode at acidic, neutral and basic environment (pH 5, 7 and 9) over 45 days. After each measurement, the electrode was rinsed and stored in a storage box until the next testing cycle. As shown in Fig. 5c, the electrode maintained a stable pH dependent response throughout the 45 days period. To quantify the stability, the calibration slope was estimated from the OCP values measured at pH 5, 7 and 9 at each testing time. The sensitivity decreased only slightly from the first measurement day to day 45, with a sensitivity retention of approximately 96.3%. This result indicates good long-term stability of the IrO_2_/PANI/CFC sensing interface during repeated measurement and storage cycles. Finally, the practical applicability of the electrode was assessed using real samples, including vinegar, orange juice and baking powder solution. The OCP values measured by the electrode shown in Fig. S6 and the corresponding pH values were then calculated from the standard calibration curve of the developed sensor. The calculated pH values were then compared with those obtained using a commercial glass membrane pH meter (EZDO PH5011) under the same conditions. As shown in Fig. 5d, the commercial pH meter measured pH values of 2.5, 3.5 and 8.3 for vinegar, orange juice, and baking powder solution, respectively, whereas the IrO_2_/PANI/CFC electrode gave corresponding values of 2.6, 3.3 and 8.2.

The close agreement between the two methods confirms that the flexible IrO_2_/PANI/CFC electrode can provide reliable pH measurements in both standard buffer solutions and practical sample environments. The performance of the IrO_2_/PANI/CFC electrode was further compared with recently reported PANI, IrO_*x*_ and PANI/oxide-based potentiometric pH sensors, as summarized in Table S1. The present electrode showed sensitivity comparable to several single-component PANI or IrO_*x*_ based electrodes and higher than reported PANI/metal oxide hybrid electrodes, while also maintaining 96.3% sensitivity retention after 45 days.

## 4 Conclusions

In summary, this study demonstrates that direct integration of an IrO_2_/PANI bilayer onto conductive carbon fibre cloth is an effective strategy for developing a binder free solid-state potentiometric pH sensor. SEM, TEM, EDX, FTIR, Raman, and XPS analyses verified the formation of a hybrid sensing layer on the carbon fibre network, confirming that the sequential modification process produced a chemically and structurally distinct sensing interface rather than a simple carbon substrate coating. The resulting IrO_2_/PANI/CFC electrode showed a stable, linear, and near-Nernstian open-circuit potential response over pH 3–11 and maintained comparable pH sensitivity during continuous pH adjustment, indicating that the electrode can operate under both stepwise calibration and dynamic measurement conditions. The limited response toward common cations, reversible pH tracking behavior, acceptable short-term drift and 96.3% sensitivity retention after 45 days further support the operational stability of the bilayer interface. Real sample measurements in acidic and alkaline samples were consistent with a commercial glass pH meter, demonstrating the practical relevance of the sensor beyond standard buffer solutions. Overall, the IrO_2_/PANI/CFC architecture provides a simple, low-cost, and stable platform for solid-state pH sensing in aqueous sample analysis. Further development of this platform should include systematic evaluation under mechanical deformation including bending, cyclic strain and conformal contact with irregular surfaces, to establish its suitability for wearable and curved surface pH sensing formats.

## Ethical statement

The authors confirm that this research did not involve human participants, human samples, live animals, or any other studies requiring institutional ethical approval. Therefore, ethical approval was not obtained for this study.

## Author contributions

Md Rasel: conceptualization, methodology, investigation, data collection, formal analysis, visualization, and writing—original draft preparation. Mamun Jamal: conceptualization, visualization, supervision, validation, writing, review, and editing (lead). N. Padmanathan: characterization, validation, writing—review and editing. Kafil M. Razeeb: writing—review and editing. All authors reviewed and approved the final version of the manuscript and agreed to be accountable for all aspects of this work.

## Conflicts of interest

The authors declare that they have no competing interests.

## Supplementary Material

RA-OLF-D6RA05424B-s001

## Data Availability

All data supporting the findings of this study are included in the main manuscript and/or in the supplementary information (SI). The SI contains the relevant experimental data, characterization results, and SI used for the analysis and interpretation. No additional code or software is required beyond the standard data analysis tools. Supplementary information is available. See DOI: https://doi.org/10.1039/d6ra05424b.

## References

[cit1] Wachta I., Balasubramanian K. (2024). Electroanalytical Strategies for Local pH Sensing at Solid–Liquid Interfaces and Biointerfaces. ACS Sens..

[cit2] Manjakkal L., Szwagierczak D., Dahiya R. (2020). Metal oxides based electrochemical pH sensors: Current progress and future perspectives. Prog. Mater. Sci..

[cit3] Ghoneim M. T. (2019). *et al.*, Recent Progress in Electrochemical pH-Sensing Materials and Configurations for Biomedical Applications. Chem. Rev..

[cit4] Sheet P. S., Park S., Nguyen A. T., George S., Maier C., Koley D. (2024). Triple-function carbon-based Ca2+ ion-selective pH ring microelectrode to study real-time bacteria-mediated hydroxyapatite corrosion. Anal. Chim. Acta.

[cit5] Manjakkal L., Dervin S., Dahiya R. (2020). Flexible potentiometric pH sensors for wearable systems. RSC Adv..

[cit6] Mariani F. (2020). *et al.*, Design of an electrochemically gated organic semiconductor for pH sensing. Electrochem. Commun..

[cit7] Chen Y. (2025). *et al.*, Utilizing Hydrogel-Isolated Dual-Carboxyl Surface-Enhanced Raman Scattering Probe for Accurate Online pH Monitoring. ACS Sens..

[cit8] Bi L. (2018). *et al.*, Highly Sensitive and Reproducible SERS Sensor for Biological pH Detection Based on a Uniform Gold Nanorod Array Platform. ACS Appl. Mater. Interfaces.

[cit9] Lu X. (2024). *et al.*, Surface enhanced Raman scattering microtips for microenvironment pH determination of semi-solid preparations. Anal. Methods.

[cit10] Wu X. (2026). *et al.*, Recent Progress of Soft and Bioactive Materials in Flexible Bioelectronics. Cyborg Bionic Syst..

[cit11] Dong M. (2026). *et al.*, High-strength, electrochemically efficient RuO2-MXene/CNTs hybrid fibres via nanostructure design for flexible electronics and energy storage. Mater. Des..

[cit12] Xie L., Liu L., Xu S., Wang T., Yue X., Li G. (2024). An efficient voltammetric sensing platform for trace determination of Norfloxacin based on nanoplate-like α-zirconium phosphate/carboxylated multiwalled carbon nanotube nanocomposites. Microchem. J..

[cit13] Li J., Li L., Fei J., Zhao P., zhao J., Xie Y. (2024). Ultrasensitive electrochemical sensor for fenitrothion based on MIL-125 derived iron/titanium bimetallic oxides doped porous carbon composite. Microchem. J..

[cit14] Marques M. J. M. (2026). *et al.*, Bioinspired Microtexturing for Enhanced Sweat Adhesion in Ion-Selective Membranes. Cyborg Bionic Syst..

[cit15] Jamal M. (2019). *et al.*, Development of Tungsten Oxide Nanoparticle Modified Carbon Fibre Cloth as Flexible pH Sensor. Sci. Rep..

[cit16] Pattan-Siddappa G., Elugoke S. E., Erkmen C., Kim S.-Y., Ebenso E. E. (2025). Flexible carbon cloth electrode: pioneering the future of electrochemical sensing devices. Adv. Compos. Hybrid Mater..

[cit17] Pedaballi S., Li C.-C. (2022). Using conductive carbon fabric to fabricate binder-free Ni-rich cathodes for Li-ion batteries. Int. J. Energy Res..

[cit18] Rasel M., Teixeira S. R., Islam J., Hamidi H., Quinn A. J., Iacopino D. (2026). Laser-Induced Graphene-Based Potentiometric pH and Nonenzymatic Uric Acid Sensors for Urine Analysis on Baby Diaper. ACS Omega.

[cit19] Li Y., Mao Y., Xiao C., Xu X., Li X. (2020). Flexible pH sensor based on a conductive PANI membrane for pH monitoring. RSC Adv..

[cit20] Hossain M. S., Padmanathan N., Badal M. M. R., Razeeb K. M., Jamal M. (2024). Highly Sensitive Potentiometric pH Sensor Based on Polyaniline Modified Carbon Fibre Cloth for Food and Pharmaceutical Applications. ACS Omega.

[cit21] Park H. J., Yoon J. H., Lee K. G., Choi B. G. (2019). Potentiometric performance of flexible pH sensor based on polyaniline nanofibre arrays. Nano Converg..

[cit22] Zhang J., Salehirozveh M., Rahimi S., Gerner E., Pandit S., Mijakovic I. (2026). Electrochemically Synthesized Polyaniline Nanofibres on Flexible Electrode for pH Sensing. Electroanalysis.

[cit23] Nur-E-Alam M. (2023). *et al.*, Physical-Vapor-Deposited Metal Oxide Thin Films for pH Sensing Applications: Last Decade of Research Progress. Sensors.

[cit24] Shylendra S. P., Wajrak M., Kang J. J. (2025). Advancements in Solid-State Metal pH Sensors: A Comprehensive Review of Metal Oxides and Nitrides for Enhanced Chemical Sensing: A Review. IEEE Sens. J..

[cit25] Liu J., Han C., Chen J., Nan L., Si Y. (2024). An All-Solid-State Ti/RuOx pH Electrode Prepared Based on the Thermal Oxidation Method. ACS Sens..

[cit26] Madhu S., Ramasamy S., Choi J. (2026). Wireless IrO2 Electrochemical Sensor System for Real-Time pH Monitoring in Microliter Volumes. ACS Meas. Sci. Au.

[cit27] Sun Z., Ma Q., Wang Y., Pan Y. (2020). Effects of Structures on the Sensing Properties of Long-Term Stable IrOx pH Electrodes. J. Electrochem. Soc..

[cit28] Chou S.-C. (2022). *et al.*, A flexible IrO2 membrane for pH sensing. Sci. Rep..

[cit29] Dakshayini B. S. (2019). *et al.*, Role of conducting polymer and metal oxide-based hybrids for applications in ampereometric sensors and biosensors. Microchem. J..

[cit30] Akhtar M., Shahzadi S., Arshad M., Akhtar T., Saeed Ashraf Janjua M. R. (2025). Metal oxide-polymer hybrid composites: a comprehensive review on synthesis and multifunctional applications. RSC Adv..

[cit31] Islam M., Hossain M. S., Padmanathan N., Razeeb K. M., Jamal M. (2025). A highly sensitive and reliable pH sensor based on a polyaniline-nickel hydroxide modified nickel foam electrode: electrochemical and DFT investigations. Mater. Adv..

[cit32] Lee J., Soltis I., Tillery S. A., Lee S. H., Kim H., Yeo W.-H. (2024). Long-term stable pH sensor array with synergistic bilayer structure for 2D real-time mapping in cell culture monitoring. Biosens. Bioelectron..

[cit33] Zhao Y., Hernandez-Pagan E. A., Vargas-Barbosa N. M., Dysart J. L., Mallouk T. E. (2011). A High Yield Synthesis of Ligand-Free Iridium Oxide Nanoparticles with High Electrocatalytic Activity. J. Phys. Chem. Lett..

[cit34] Wickramasinghe D., Oh J.-M., McGraw S., Senecal K., Chow K.-F. (2019). Electrochemical Effects of Depositing Iridium Oxide Nanoparticles onto Conductive Woven and Nonwoven Flexible Substrates. ACS Appl. Energy Mater..

[cit35] Zhao Y., Vargas-Barbosa N. M., Hernandez-Pagan E. A., Mallouk T. E. (2011). Anodic Deposition of Colloidal Iridium Oxide Thin Films from Hexahydroxyiridate(IV) Solutions. Small.

[cit36] Geniès E. M., Boyle A., Lapkowski M., Tsintavis C. (1990). Polyaniline: A historical survey. Synth. Met..

[cit37] Jamadade V. S., Dhawale D. S., Lokhande C. D. (2010). Studies on electrosynthesized leucoemeraldine, emeraldine and pernigraniline forms of polyaniline films and their supercapacitive behavior. Synth. Met..

[cit38] Tarver J., Yoo J. E., Dennes T. J., Schwartz J., Loo Y.-L. (2009). Polymer Acid Doped Polyaniline Is Electrochemically Stable Beyond pH 9. Chem. Mater..

[cit39] Zhu X. (2024). *et al.*, A flexible pH sensor based on polyaniline@oily polyurethane/polypropylene spunbonded nonwoven fabric. RSC Adv..

[cit40] Desimoni E., Casella G. I., Morone A., Salvi A. M. (1990). XPS determination of oxygen-containing functional groups on carbon-fibre surfaces and the cleaning of these surfaces. Surf. Interface Anal..

[cit41] Sun T., Li M., Zhou S., Liang M., Chen Y., Zou H. (2020). Multi-scale structure construction of carbon fibre surface by electrophoretic deposition and electropolymerization to enhance the interfacial strength of epoxy resin composites. Appl. Surf. Sci..

[cit42] Feng X.-M. (2011). *et al.*, One-Step Electrochemical Synthesis of Graphene/Polyaniline Composite Film and Its Applications. Adv. Funct. Mater..

[cit43] Huerta F., Quijada C., Montilla F., Morallón E. (2021). Revisiting the Redox Transitions of Polyaniline. Semiquantitative Interpretation of Electrochemically Induced IR Bands. J. Electroanal. Chem..

[cit44] Trchová M., Stejskal J. (2011). Polyaniline: The infrared spectroscopy of conducting polymer nanotubes (IUPAC Technical report). Pure Appl. Chem..

[cit45] Okuda H., Young R. J., Wolverson D., Tanaka F., Yamamoto G., Okabe T. (2018). Investigating nanostructures in carbon fibres using Raman spectroscopy. Carbon.

[cit46] Yu P., Li Y., Yu X., Zhao X., Wu L., Zhang Q. (2013). Polyaniline Nanowire Arrays Aligned on Nitrogen-Doped Carbon Fabric for High-Performance Flexible Supercapacitors. Langmuir.

[cit47] Trchová M., Morávková Z., Bláha M., Stejskal J. (2014). Raman spectroscopy of polyaniline and oligoaniline thin films. Electrochim. Acta.

[cit48] Freakley S. J., Ruiz-Esquius J., Morgan D. J. (2017). The X-ray photoelectron spectra of Ir, IrO2 and IrCl3 revisited. Surf. Interface Anal..

[cit49] Roiron C., Wang C., V Zenyuk I., Atanassov P. (2024). Oxygen 1s X-ray Photoelectron Spectra of Iridium Oxides as a Descriptor of the Amorphous–Rutile Character of the Surface. J. Phys. Chem. Lett..

[cit50] Idriss H. (2021). On the wrong assignment of the XPS O1s signal at 531–532 eV attributed to oxygen vacancies in photo- and electro-catalysts for water splitting and other materials applications. Surf. Sci..

[cit51] Chen Y., Kang E. T., Neoh K. G., Lim S. L., Ma Z. H., Tan K. L. (2001). Intrinsic redox states of polyaniline studied by high-resolution X-ray photoelectron spectroscopy. Colloid Polym. Sci..

[cit52] Kang E. T., Neoh K. G., Khor S. H., Tan K. L., Tan B. T. G. (1989). Structural determination of polyaniline by X-ray photoelectron spectroscopy. J. Chem. Soc. Chem. Commun..

[cit53] Mariani F. (2021). *et al.*, Advanced Wound Dressing for Real-Time pH Monitoring. ACS Sens..

[cit54] Kinlen P. J., Heider J. E., Hubbard D. E. (1994). A solid-state pH sensor based on a Nafion-coated iridium oxide indicator electrode and a polymer-based silver chloride reference electrode. Sens. Actuators, B.

