# Supplementary material for: Carbon fibre cloth-supported iridium oxide/polyaniline bilayer electrode for solid-state potentiometric pH sensing
Source: RSC Adv. 2026 Jul 23;16(38):42528–39. doi: 10.1039/d6ra05424b (PMC13392754; doi:10.1039/d6ra05424b)
Supplement: Supplementary file 1 [file RA-016-D6RA05424B-s001.pdf]

Supplementary Information (SI) for RSC Advances.
This journal is © The Royal Society of Chemistry 2026

Supporting Information

Carbon Fibre Cloth-Supported Iridium Oxide/Polyaniline Bilayer Electrode for Solid-State Potentiometric pH Sensing

Md Rasel[a], Mamun Jamal[a*], N. Padmanathan[b], Kafil M. Razeeb[b*]

[a]Department of Chemistry, Khulna University of Engineering & Technology, Khulna 9203, Bangladesh

[b]Micro-NanoSystems Centre, Tyndall National Institute, University College Cork, Lee Maltings Complex, Dyke Parade, Cork T12 R5CP, Ireland

[*]Corresponding authors Email: mamun.jamal@chem.kuet.ac.bd; kafil.mahmood@tyndall.ie

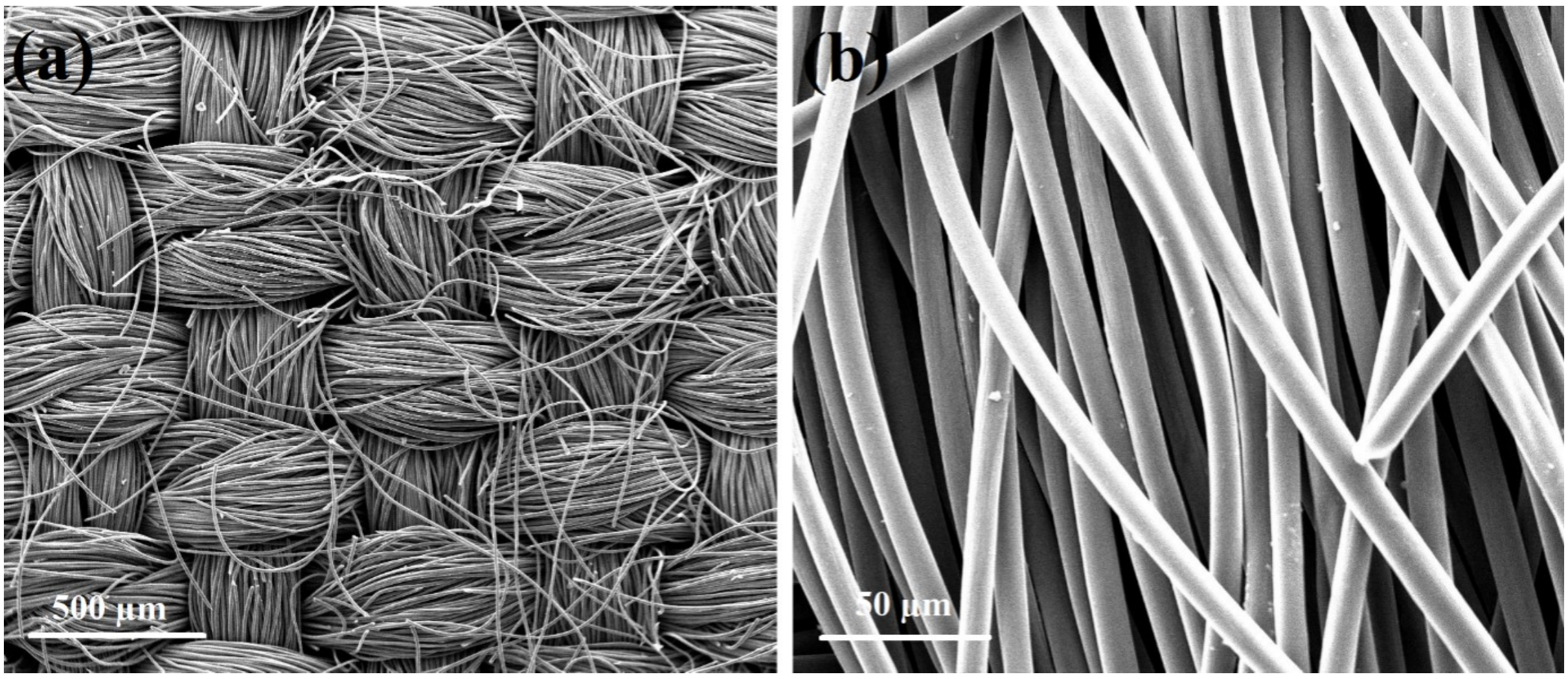

Fig. S1. SEM images of bare CFC at different magnifications.

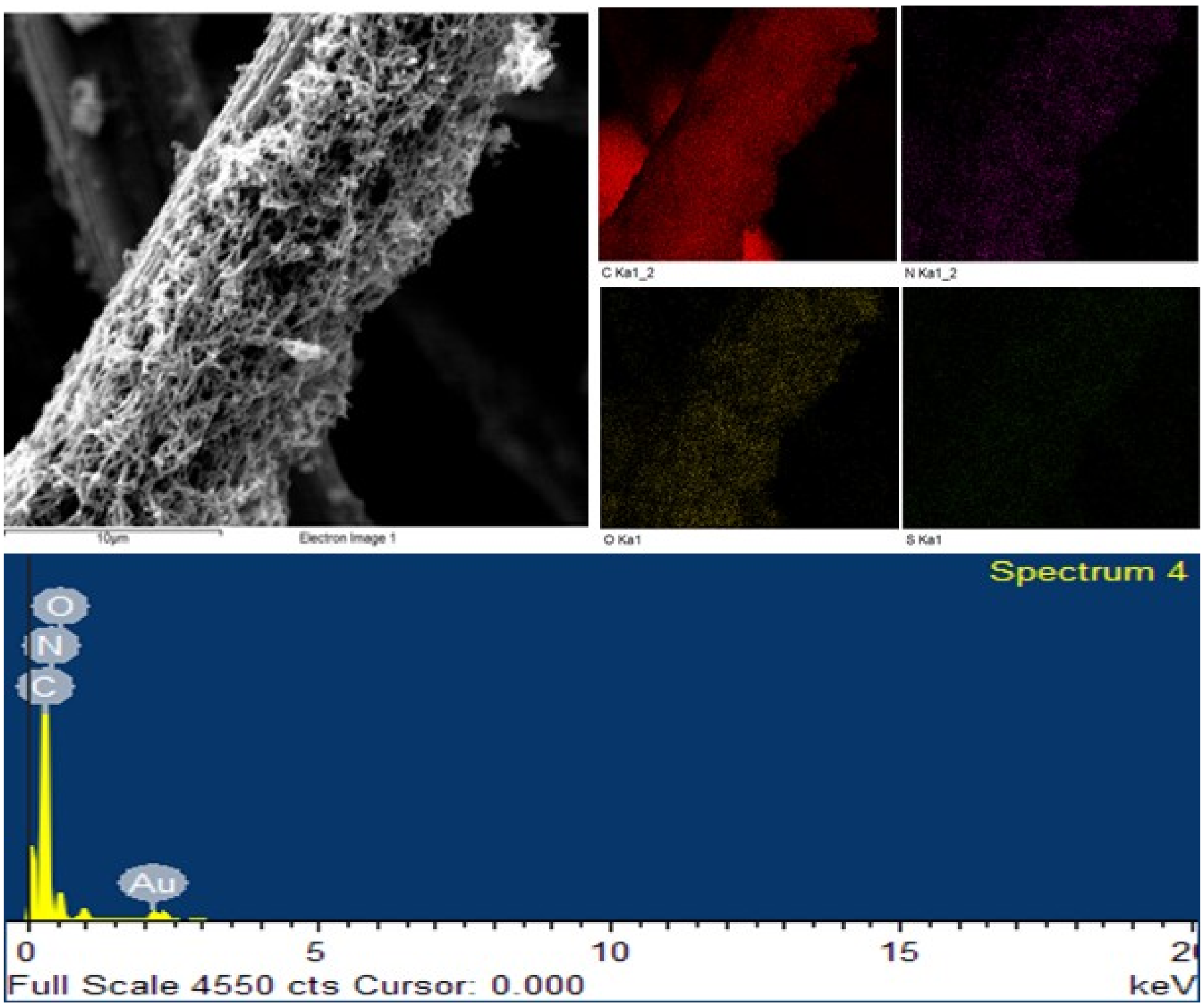

Fig. S2. EDX mapping of PANI modified CFC electrode.

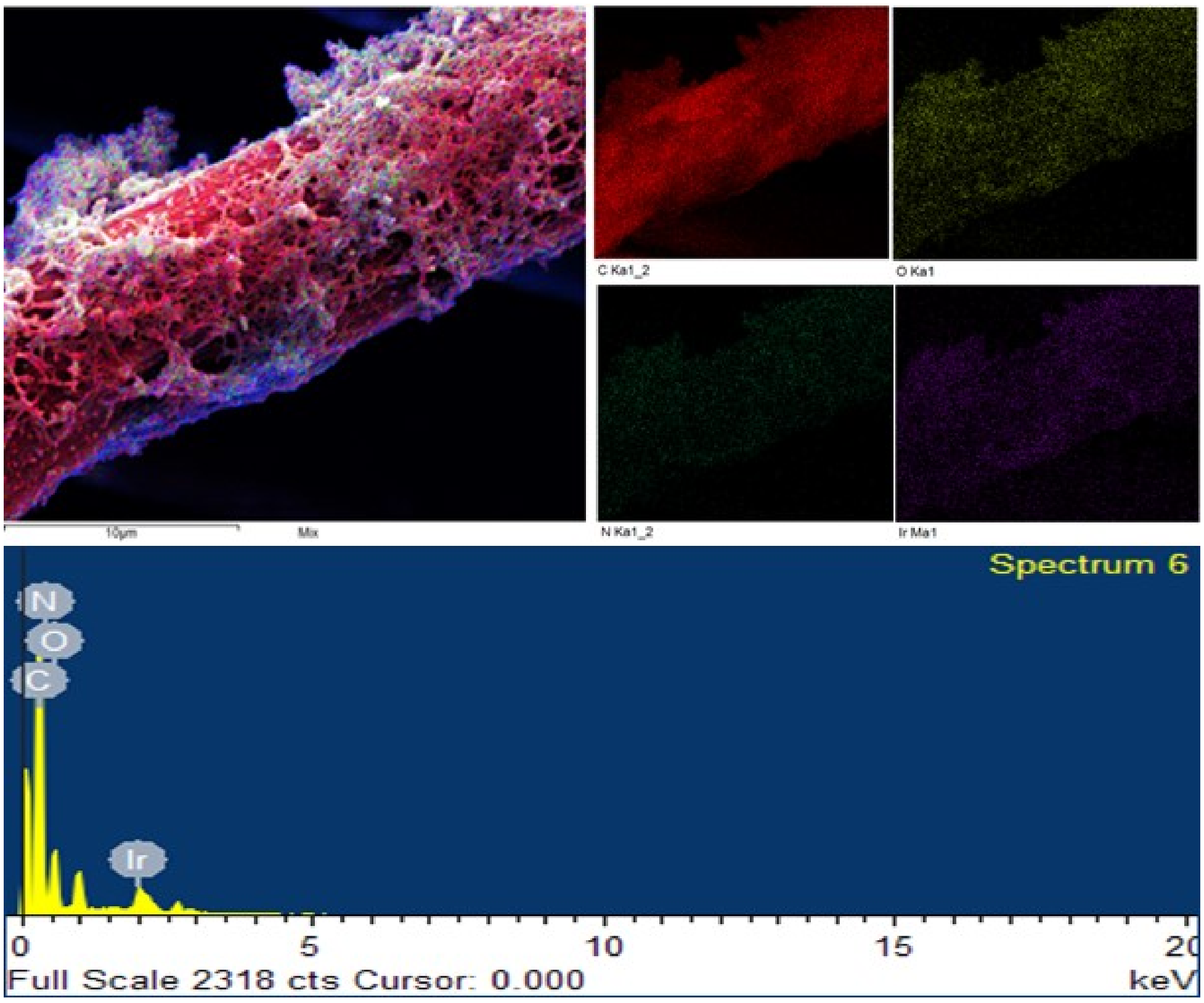

Fig. S3. EDX mapping of IrO_2-PANI/CFC electrode.

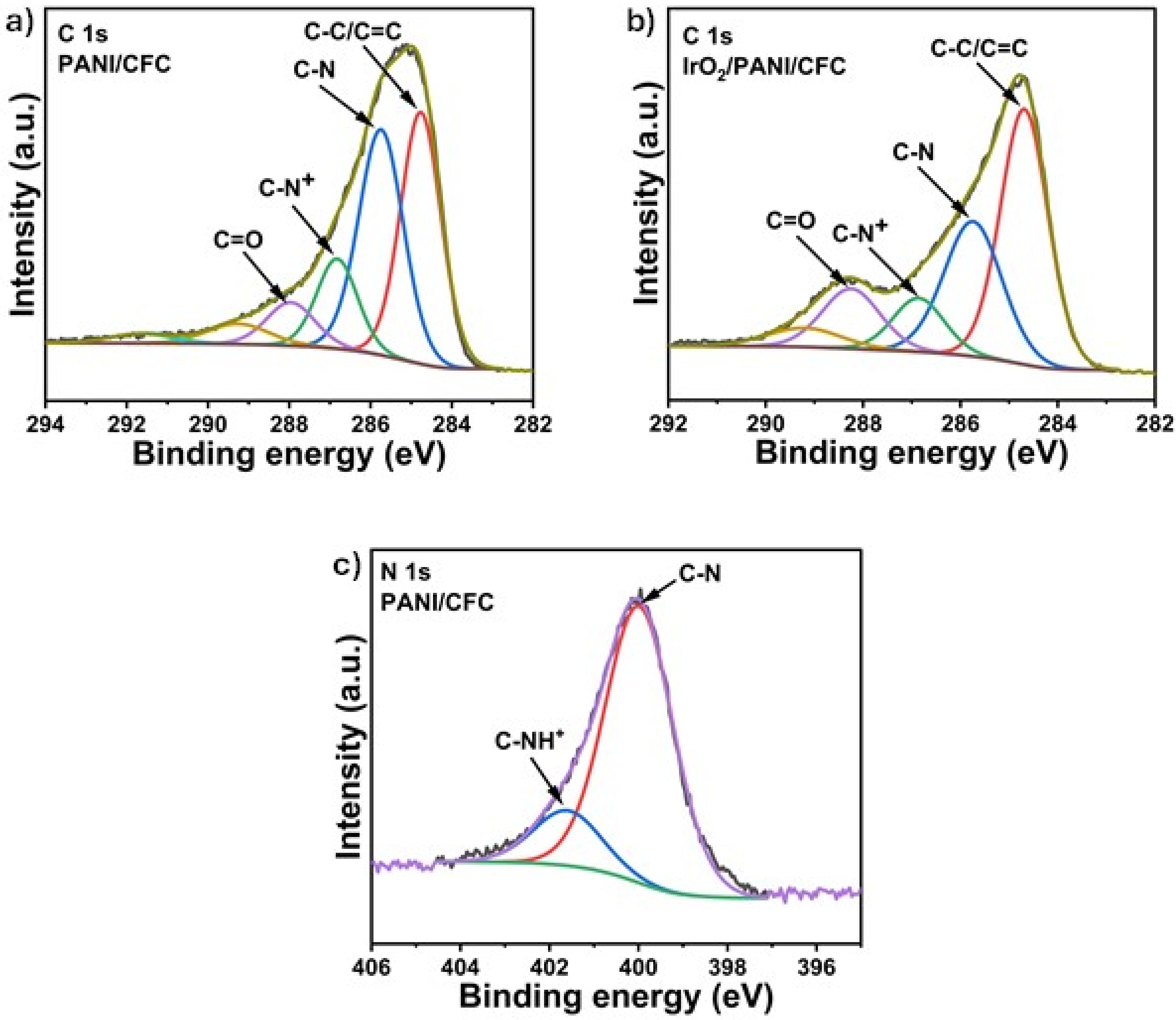

Fig. S4. Additional high-resolution XPS fitting profiles of the modified electrodes. (a, b) C 1s spectra of PANI/CFC and IrO_2/PANI/CFC, respectively; (c) N 1s spectra of PANI/CFC showing neutral C–N and protonated C–NH^+ nitrogen species associated with the PANI backbone.

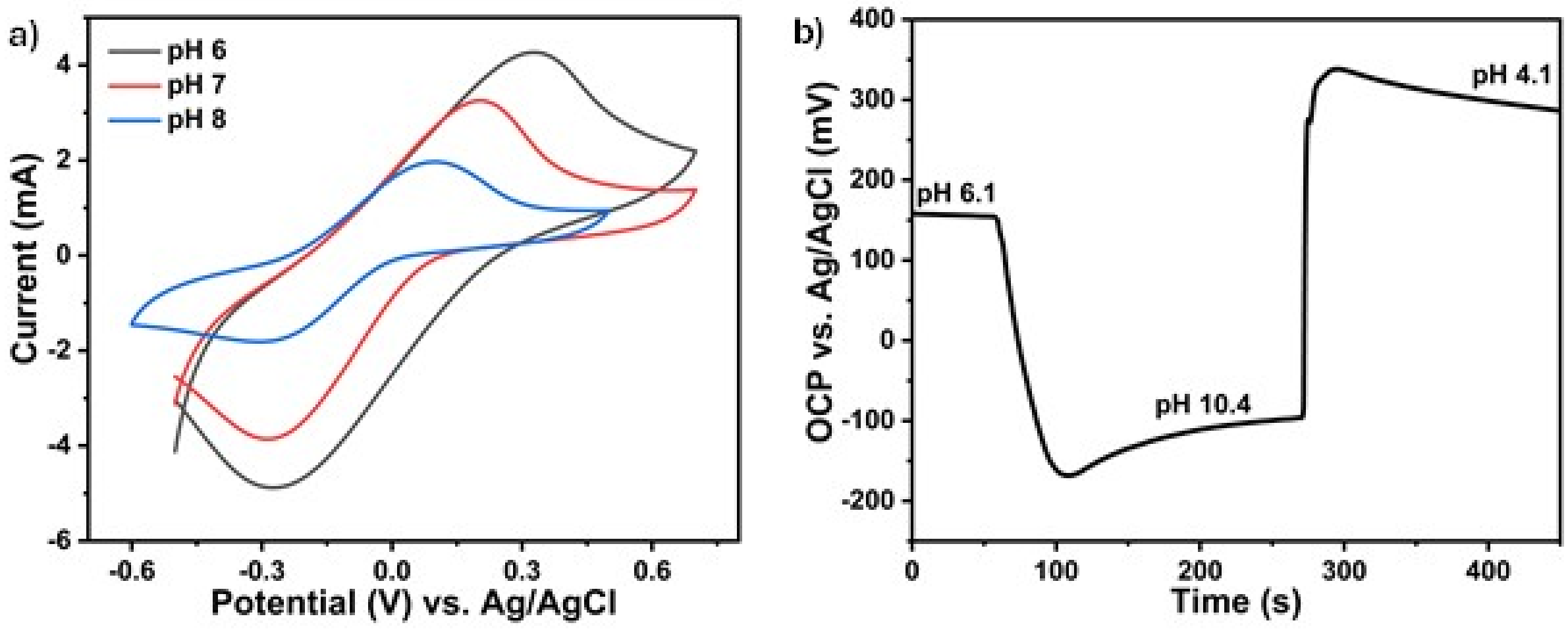

Fig. S5. (a) Cyclic voltammograms of IrO_2/PANI/CFC electrode at different pH buffer conditions (acidic, neutral and basic); (b) Reversibility test of IrO_2/PANI/CFC electrode in acidic and basic medium.

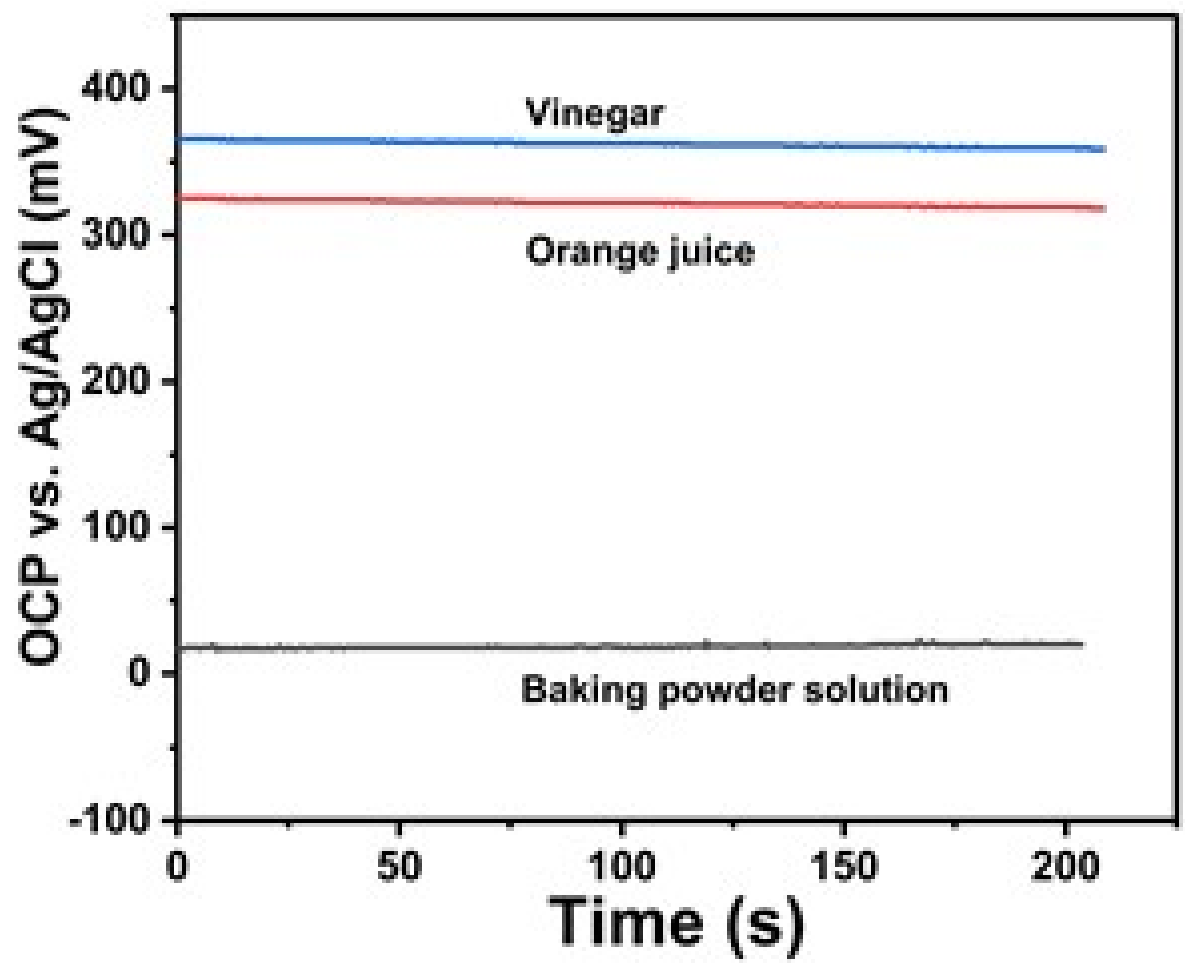

Fig. S6. OCP response of the IrO_2/PANI/CFC electrode in real samples (vinegar, orange juice and baking powder solution).

Table S1. Comparison of the sensing performance of the IrO_2/PANI/CFC electrode with previously reported IrO_x, PANI based potentiometric pH sensors.[a]

Electrode Materials	Substrate	pH range	Sensitivity (mV/pH)	Drift	Long-term stability (days)	Real sample	Ref.
PANI-Au NWs	PDMS	4-8	56.1	~ 1.8 mV/h	-	Sweat	[1]
PANI	CFC	4-12	60.9	10 mV/3h	-	Food	[2]
PANI	LIG	4-10	56.7 ± 2.6	8.4 mV/h	42	Ar. urine	[3]
INA	PNA	4-10	69.43	0.76 mV/h	-	Sweat	[4]
PANI-Au	PET	3-8	63.7	0.7 mV/h	-	Sweat	[5]
IrO_x	PI	2-13	60.6	10.5 mV	-	Ar. sweat	[6]
IrO_x	Si-Si_3N_4	1.8-12	82.88	-	32	Tap water	[7]
PANI/$Ni(OH)_2$	NF	3-11	46	2.34 mV/h	-	Food	[8]
PANI-IrO_x	PI/Cu/Au	3-10	40.1 ± 0.5	-	60	Cell culture	[9]
IrO_2/PANI	CFC	3-11	58.4 ± 0.9	~ 9 mV/6h	45	Food	**This work**

[a]PANI-Au NWs, polyaniline gold nanowires; PDMS, polydimethylsiloxane; LIG, Laser-Induced graphene; PNA, polyurethane acrylate nanopillar array; INA, IrO_x-Au nanopillars array; PI, Polyimide; NF, nickel foam.

References

[1] Q. Zhai *et al.*, "Vertically Aligned Gold Nanowires as Stretchable and Wearable Epidermal Ion-Selective Electrode for Noninvasive Multiplexed Sweat Analysis," *Anal. Chem.*, vol. 92, no. 6, pp. 4647–4655, Mar. 2020, doi: 10.1021/acs.analchem.0c00274.

[2] M. S. Hossain, N. Padmanathan, M. M. R. Badal, K. M. Razeeb, and M. Jamal, "Highly Sensitive Potentiometric pH Sensor Based on Polyaniline Modified Carbon Fibre Cloth for Food and Pharmaceutical Applications," *ACS Omega*, vol. 9, no. 38, pp. 40122–40133, Sep. 2024, doi: 10.1021/acsomega.4c06090.

[3] M. Rasel, S. R. Teixeira, J. Islam, H. Hamidi, A. J. Quinn, and D. Iacopino, "Laser-Induced Graphene-Based Potentiometric pH and Nonenzymatic Uric Acid Sensors for Urine Analysis on Baby Diaper," *ACS Omega*, vol. 11, no. 17, pp. 25139–25148, May 2026, doi: 10.1021/acsomega.5c11146.

[4] E. S. Yoon, H. J. Park, M. S. Kil, J. Kim, K. G. Lee, and B. G. Choi, "Preparation of nanopillar array electrode of iridium oxide for high performance of pH sensor and its real-time sweat monitoring," *Bull. Korean Chem. Soc.*, vol. 44, no. 6, pp. 528–535, Jun. 2023, doi: https://doi.org/10.1002/bkcs.12689.

[5] H. Y. Y. Nyein *et al.*, "A Wearable Electrochemical Platform for Noninvasive Simultaneous Monitoring of Ca2+ and pH," *ACS Nano*, vol. 10, no. 7, pp. 7216–7224, Jul. 2016, doi: 10.1021/acsnano.6b04005.

[6] K. Chawang, S. Bing, and J.-C. Chiao, "Printable and Flexible Iridium Oxide-Based pH Sensor by a Roll-to-Roll Process," 2023. doi: 10.3390/chemosensors11050267.

[7] J. Yin, W. Chen, W. Gao, X. Zhang, B. Tang, and Q. Jin, "Batch Fabrication of Microminiaturized pH Sensor Integrated With IrOx Film and Solid State Ag/AgCl Electrode for Tap Water Quality Online Detection," *IEEE Sens. J.*, vol. 23, no. 4, pp. 3475–3484, 2023, doi: 10.1109/JSEN.2023.3234140.

[8] M. Islam, M. S. Hossain, N. Padmanathan, K. M. Razeeb, and M. Jamal, "A highly sensitive and reliable pH sensor based on a polyaniline-nickel hydroxide modified nickel foam electrode: electrochemical and DFT investigations," *Mater. Adv.*, vol. 6, no. 21, pp. 7895–7905, Nov. 2025, doi: 10.1039/d5ma00830a.

[9] J. Lee, I. Soltis, S. A. Tillery, S. H. Lee, H. Kim, and W.-H. Yeo, "Long-term stable pH sensor array with synergistic bilayer structure for 2D real-time mapping in cell culture monitoring," *Biosens. Bioelectron.*, vol. 254, p. 116223, 2024, doi: https://doi.org/10.1016/j.bios.2024.116223.
